# Crosstalk Between the Endoplasmic Reticulum and Mitochondria in Skeletal Muscle: Implications for Meat Quality

**DOI:** 10.3390/ani15233465

**Published:** 2025-12-01

**Authors:** Wenjin Peng, Yiting Guo, Xiaolin Wu, Liuteng Miao, Xihong Zhou

**Affiliations:** 1 College of Animal Science and Technology, Hunan Agricultural University, Changsha 410128, China; 2 Institute of Subtropical Agriculture, Chinese Academy of Sciences, Changsha 410125, China

**Keywords:** mitochondria, endoplasmic reticulum, mitochondria-associated membranes, meat quality

## Abstract

The coordination between the endoplasmic reticulum (ER) and mitochondria is increasingly recognized as a central regulator of skeletal muscle metabolism and, consequently, meat quality. Here, we summarize recent progress on how ER–mitochondrial communication regulates muscle metabolism and meat-quality traits. This interaction, primarily mediated by mitochondria-associated membranes, integrates key cellular processes, including calcium signaling, redox homeostasis, lipid metabolism, and cellular stress responses. These events then lead to tissue-level outcomes such as intramuscular fat deposition, transitions in muscle fiber types, and postmortem apoptotic or proteolytic events, all of which influence meat quality. However, comparative studies across livestock species reveal notable gaps and inconsistencies, highlighting the need for deeper mechanistic investigation. Overall, deepening our understanding of ER–mitochondria crosstalk will inform nutritional or molecular strategies to enhance meat quality and clarify species-specific regulatory mechanisms.

## Simple Summary

Meat quality, defined by various traits including tenderness, flavor, and fat content, is largely determined by the development and function of skeletal muscle cells. The coordination between the endoplasmic reticulum and mitochondria is central to muscle growth and energy homeostasis in muscle cells. This review summarizes how ER–mitochondria crosstalk shapes meat quality traits by regulating energy production, fiber assembly and fat storage, and how disruptions in this interplay compromise muscle function after slaughter. A clearer understanding of these organelle interactions offers valuable insights to improve meat production and quality in animal husbandry and the food industry.

## 1. Introduction

### 1.1. Structure and Basic Function of Mitochondria and Endoplasmic Reticulum

Mitochondria are thought to have originated from alpha-proteobacterium through an ancient endosymbiotic event. Their primary function of these dynamic, double-membraned organelles is to provide energy for cells. Structurally, mitochondria are enclosed by a double-membrane system composed of the outer mitochondrial membrane (OMM) and the inner mitochondrial membrane (IMM), which together define the intermembrane space (IMS) and surround the mitochondrial matrix (MM) [1].

Mitochondria possess a highly compartmentalized structure that enables diverse metabolic functions. The OMM is a smooth, lipid-rich membrane containing β–barrel proteins such as voltage–dependent anion-selective channels or porins, as well as α–helical proteins including mitochondrial fission factor and mitochondrial Rho GTPase, which serve as receptors involved in mitochondrial division and motility. The IMM, the most protein–rich membrane in eukaryotic cells, is partitioned into the inner boundary membrane (IBM) and the cristae. The IBM runs parallel to the OMM and is enriched with membrane transport proteins that allows ion exchange between the cytoplasm and the mitochondrial matrix. Cristae, formed by inward invaginations of the IMM, create specialized regions enriched in the oxidative phosphorylation system and the electron transport chain [2]. The MM hosts the mitochondrial genetic system, numerous biosynthetic processes, and the tricarboxylic acid (TCA) cycle, which generates NADH and FADH_2_ to fuel ATP synthesis through chemiosmotic energy [3]. The IMS is the smallest mitochondrial compartment and contains only about 5% of the mitochondrial proteome, but it plays vital roles in protein and lipid synthesis, ion and redox balance, oxidative protein folding, programmed cell death, and the maintenance of mitochondrial structure and dynamics [4].

**Figure 1:**
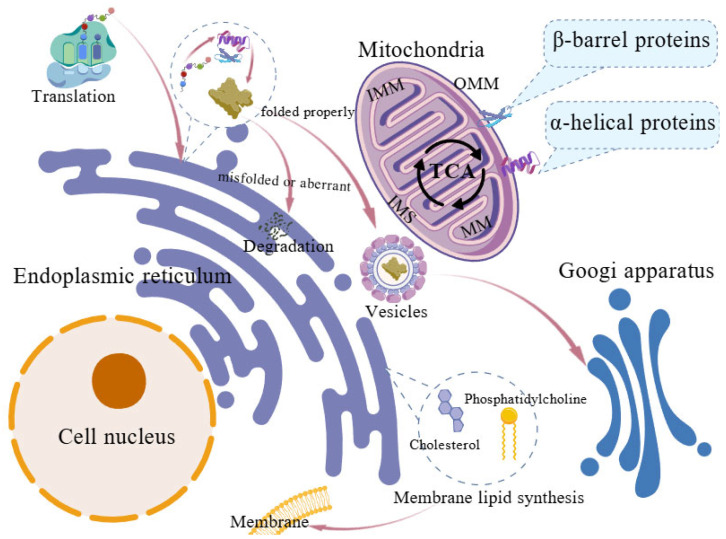
Structural organization and basic functions of the endoplasmic reticulum and mitochondria. Mitochondria, the location of the tricarboxylic acid (TCA) cycle, consist of the outer mitochondrial membrane (OMM), inner mitochondrial membrane (IMM), mitochondrial matrix (MM) and the intermembrane space (IMS). In the endoplasmic reticulum, nascent polypeptides undergo post-translational modifications and chaperone–mediated folding, with correctly folded proteins packaged into COPII vesicles for Golgi transport. Misfolded proteins are removed via ER–associated degradation. Major membrane lipids are synthesized in the ER, including phosphatidylcholine, phosphatidylethanolamine, and cholesterol.

The endoplasmic reticulum (ER) is a multifunctional organelle that maintains cellular homeostasis by coordinating protein folding, calcium signaling, redox regulation, and lipid biosynthesis. It comprises roughly 60% of the total cellular membrane system [5]. Functionally, nearly one-third of the human proteome is synthesized in the ER, where nascent polypeptides undergo essential post-translational modifications and chaperone-mediated folding to achieve their native conformations [6]. Properly folded proteins are subsequently packaged into coat protein complex II–coated vesicles for transport to the Golgi apparatus, whereas misfolded or aberrant proteins are recognized and eliminated via ER-associated degradation. Beyond protein maturation, the ER is the major site of membrane-lipid biogenesis, producing phosphatidylcholine, phosphatidylethanolamine, and cholesterol, which are fundamental composition for biological membrane. The ER membrane is enriched in glycerophospholipids such as phosphatidic acid, facilitating inter–organelle communication and rapid modulation of membrane dynamics. Disruptions of ER homeostasis compromise these critical functions, contributing to diverse pathological conditions (Figure 1) [6].

The ER and mitochondria engage in extensive functional coupling that coordinates cellular metabolism. Many ER–dependent processes require ATP generated by mitochondria, whereas mitochondria rely on the ER for the supply of specific lipids and biosynthetic precursors required for maintaining structural integrity and functions. Mitochondrial dynamics, including fission and fusion, are tightly regulated by ER–associated molecules such as actin filament [7,8]. Additionally, both organelles act as major intracellular calcium reservoirs and coordinate closely to maintain cellular calcium homeostasis [9].

### 1.2. Functional Crosstalk Between Mitochondria and ER

Mitochondria and the ER form specialized contact sites that enable direct communication between the two organelles, known as mitochondria-associated membranes (MAMs) or mitochondria–ER contacts (MERCs) [10,11]. These structures play a crucial role in regulating calcium homeostasis, lipid homeostasis, cell survival, inflammation, ER stress (ERS) and other essential cellular functions [12].

**Figure 2:**
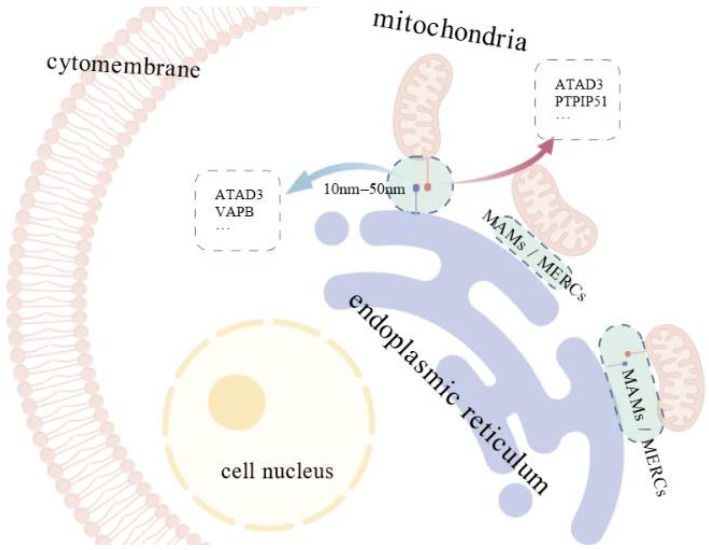
Mitochondria–ER contact sites and associated tethering proteins. Mitochondria–associated membranes (MAMs), also called mitochondria-ER contacts (MERCs), are regions where the membranes of the endoplasmic reticulum (ER) and mitochondria come into close proximity. The width of the MAM region is approximately 10–50 nm, and the two organelles are connected by tethering proteins, including ATAD3, VAPB and PTPIP51, anchored in their membranes.

MAMs are regions where the membranes of the ER and mitochondria come into close proximity without fusing, enabling each organelle to maintain its distinct identity [13]. The width of the MAM region is approximately 10–50 nm, and the two organelles are connected by tethering proteins anchored in both membranes [14]. For instance, ATPase family ATPases associated with diverse cellular activities (AAA) domain–containing protein 3 (ATAD3) acts as a linker between the ER and mitochondria [15]. The vesicle-associated membrane protein B (VAPB) on the ERs interacts with the tyrosine phosphatase-interacting protein–51 (PTPIP51) on the OMM to assemble a tethering complex at the MAM (Figure 2) [16]. Proteomic analyses using mass spectrometry have now identified more than 1000 proteins involved in maintaining the structure and function of the MAM across human and animal models [17].

The earliest identified function of MAMs was their involvement in phospholipid synthesis and trafficking [18]. In 1998, the MAM was also shown to mediate local calcium transfer from the ER to the mitochondria [19,20]. Beyond these functions, increasing evidence now indicates that MAMs also contribute to a wide range of physiological processes, including stress responses [21], cell death [22], and lipid metabolism [23].

#### 1.2.1. ER-Mitochondrial System and Calcium Transport

**Figure 3:**
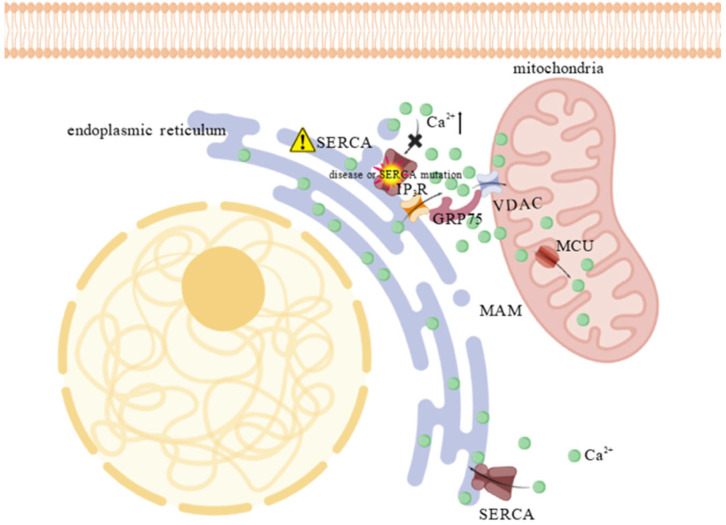
Calcium accumulation at MAM due to SERCA impairment. SERCA dysfunction (indicated by the “⚠”), caused by disease or mutation, leads to an accumulation of calcium in the cytosol, particularly at the MAM, creating a calcium–rich microdomain. The high calcium concentration activates the “calcium bridge”—a protein complex composed of IP_3_R, GRP75, and VDAC1—which channels calcium across the outer mitochondrial membrane. This rapid shuttling enables the low-affinity MCU to take up excess ions into the mitochondrial matrix, thereby buffering the cytosol and preventing calcium–induced apoptosis. The “×” indicates the blockade of calcium entry into the ER through IP_3_R and the “⬆” denotes an increase in substance concentration.

The regulation of calcium homeostasis is among the most extensively studied functions of MAMs. Calcium, a pivotal secondary messenger, is essential for numerous cellular processes [24]. Under normal conditions, the calcium level is relatively low in the cytoplasm, as the majority is sequestered in the ER by the sarco/ER calcium–ATPase (SERCA) [25,26]. Loss of ER calcium stores, usually due to disease or SERCA mutations, can trigger apoptosis. To mitigate calcium-induced apoptosis, excess cytosolic calcium is transferred to mitochondria, which possess a high buffering capacity. Calcium uptake across the OMM is mediated by voltage–dependent anion channels (VDACs), while uptake across the IMM occurs via the low–affinity mitochondrial calcium uniporter (MCU) [27,28]. Because the MCU has low calcium affinity, it requires high local calcium concentrations, which are mainly provided by the calcium–rich microdomain formed at the MAM [29]. These microdomains, located near the OMM, facilitate efficient calcium transfer from the ER to mitochondria via calcium-transfer complex [30], consisting of inositol 1,4,5–trisphosphate receptors (IP_3_Rs) on the ER, voltage–dependent anion channel 1 (VDAC1) on the OMM, and the glucose–regulated protein 75 (GRP75) chaperone that physically links them [31]. Studies have demonstrated that VDAC1 overexpression enhances mitochondrial calcium uptake, while GRP75 upregulation stabilizes IP_3_Rs–VDAC1 interactions, thereby increasing calcium influx following IP_3_R activation (Figure 3) [31,32]. Additional proteins, including VAPB in the ER membrane, also modulate calcium transport [33]. Recently, increasing studies highlight the critical role of MAM tethers in maintaining organelle communication and calcium signaling. However, whether the physical distance between membranes within MAMs directly correlates with calcium transport remains unclear and needs further investigation.

#### 1.2.2. Redox, Reactive Oxygen Species (ROS) and ER-Mitochondrial System

Beyond its role in calcium regulation, the ER–mitochondrial interface is also a crucial site for redox regulation. Notably, calcium transfer and redox signaling are functionally interconnected; for example, ROS can influence calcium signaling by oxidizing cysteine residues on IP_3_R and VDAC1, thereby modulating their open probability and affecting mitochondrial calcium uptake [34].

ROS, including superoxide (•O_2_^−^) and hydrogen peroxide (H_2_O_2_), are oxygen–derived molecules generated during mitochondrial energy metabolism and protein folding process in the ER [35]. The primary sources of ROS are mitochondria–specific complex I and III of the electron transport chain, and ER-resident oxidoreductases, such as ER oxidase–1 (ERO1). At low concentrations, ROS serve as important signaling molecules [36]; however, excessive ROS accumulation can damage cellular biomolecules, contributing to aging and the development of various diseases [37,38].

**Figure 4:**
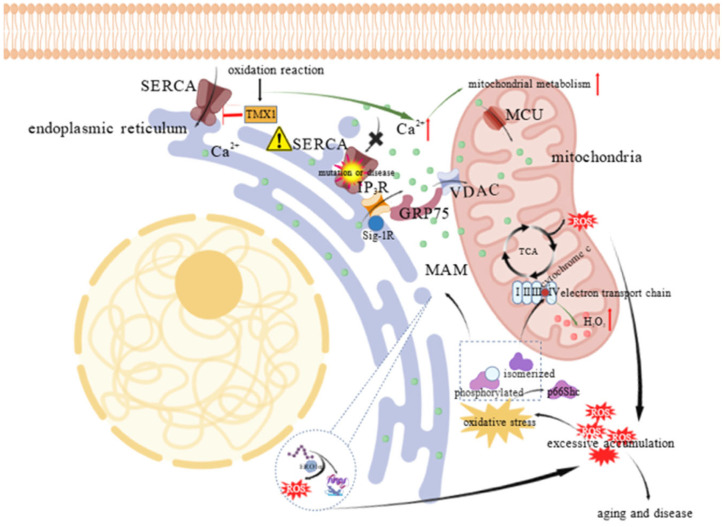
Oxidative stress induced ROS amplification and calcium regulation at mitochondria–ER contact sites. ROS are mainly generated by both mitochondrial energy metabolism and oxidative protein folding via proteins like ERO1α. Under oxidative stress, the adaptor protein p66Shc is phosphorylated, isomerized, and translocated to MAMs or mitochondria, where it interacts with cytochrome c to promote H_2_O_2_ production. In parallel, calcium signaling at this interface is tightly regulated; TMX1 inhibits SERCA in response to oxidation, increasing cytosolic calcium levels to enhance mitochondrial metabolism. This calcium transfer is further ensured by the Sig–1R, which stabilizes the IP_3_R channel for efficient calcium transit. “⬆” denotes an increase in substance concentration or metabolic level; The “⚠” indicates SERCA dysfunction; The “×” indicates the blockade of calcium entry into the ER through IP_3_R; “├” indicates inhibition.

MAMs constitute a key platform where redox signaling intersects with ER–mitochondrial communication. At MAMs, thioredoxin–related transmembrane protein 1 (TMX1) can inhibit SERCA2b activity, increasing cytosolic calcium levels and stimulating mitochondrial metabolism during the oxidative reactions [9,39]. Additionally, ERO1α generates localized H_2_O_2_ during oxidative protein folding, which can modulate adjacent redox–sensitive proteins and ion channels [40]. Under oxidative stress, the adaptor protein p66Shc is phosphorylated, isomerizes, and translocates to MAMs and/or mitochondria, where it interacts with cytochrome c to amplify H_2_O_2_ production, thereby linking stress signaling to mitochondrial redox regulation [41,42]. The Sigma–1 receptor (Sig–1R), also located at MAMs, plays a dual role: it stabilizes IP_3_Rs to facilitate ER–to–mitochondria calcium transfer, and modulates complex I activity to help maintain redox homeostasis (Figure 4) [43].

Excessive ROS production at MAMs can induce lipid peroxidation, leading to the formation of reactive aldehydes such as 4–hydroxynonenal (4–HNE), which compromise membrane integrity and disrupt signaling function [44,45]. Pathological disruption of MAM dynamics has been implicated in various diseases. For instance, in Alzheimer’s disease, the accumulation of APP/C99 at MAMs enhances ER–mitochondria tethering and ROS generation, both of which contribute to mitochondrial dysfunction [46]. Similarly, in ataxia–telangiectasia deficiency, impaired MAM tethering and calcium signaling result in defective redox regulation and mitochondrial respiratory dysfunction under stress conditions [47].

#### 1.2.3. Stress Responses and Cell Death

Cell death is a complex process that can be categorized into apoptosis, autophagy, and necrosis. The detailed molecular mechanisms of these pathways have been comprehensively described in previous studies [48,49,50] and therefore will not be reiterated here. These pathways are often activated in response to various types of stress [51]. Apoptosis, a form of programmed cell death, occurs through either intrinsic or extrinsic pathways. The intrinsic pathway is triggered by internal stressors such as mitochondrial dysfunction, DNA damage, oxidative stress, or ERS. In contrast, the extrinsic pathway is initiated after the binding of extracellular ligands to death receptors on the cell surface, which leads to the activation of caspase–8 and downstream apoptotic signaling [52]. Autophagy is primarily a survival mechanism that recycles cellular components under stress conditions, but excessive or dysregulated autophagy can also result in cell death [53]. Necrosis includes forms such as necroptosis—mediated by receptor-interacting protein kinase 1 (RIPK1), RIPK3, and mixed lineage kinase domain-like protein (MLKL) [54]—and pyroptosis, which is driven by inflammatory caspases such as caspase–1, caspase–4, or caspase-5 (and caspase–11 in mice) [55].

## 2. Investigation and Application of the ER–Mitochondrial System in Meat Quality

To synthesize the complex ER–mitochondrial signaling mechanisms discussed in this section, the major pathways and their established or hypothesized roles are summarized in Supplementary Table S1.

### 2.1. Intramuscular Fat Content

As previously discussed, the ER–mitochondrial system not only modulates energy production and storage but also mediates stress responses and maintains cellular homeostasis. In recent years, the interplay between the ER and mitochondria has emerged as a central regulator of lipid metabolism in muscle cells. Intramuscular fat (IMF), which is closely related to lipid metabolism in muscle, is a key determinant of meat quality in animal husbandry.

Multiple ER–mitochondria-associated proteins influence intramuscular lipid metabolism through distinct mitochondrial and ER pathways. These key mediators of ER–mitochondrial crosstalk include phosphatase and tensin homolog–induced kinase 1 (PINK1), carnitine palmitoyltransferase 1C (CPT1C), and cell death–inducing DNA fragmentation factor 45 (DFF45)–like effector b (CIDEB), which regulate diverse processes ranging from fatty acid oxidation to lipid droplet formation [56,57]. PINK1 controls mitochondrial quality by marking damaged mitochondria for mitophagy degradation [58], which is particularly important in energy-demanding muscle cells, where PINK1 supports efficient oxidative phosphorylation. Disruption of PINK1 signaling impairs cellular metabolism and leads to excessive fat accumulation in pig muscle fibers [59]. Studies have shown that the loss of PINK1 function in muscle cells results in the accumulation of defective mitochondria, which compromises the efficient oxidization of fatty acids [60,61]. As a result, the cellular balance shifts from lipid oxidation toward lipid storage, as cells attempt to compensate for the energy deficit caused by impaired mitochondrial function [62]. Collectively, these findings suggest that defective PINK1 disrupts the balance between lipid production and breakdown by affecting mitochondrial and ER dysfunction, thereby promoting lipid accumulation in muscle fibers and ultimately leading to an increase in IMF deposition. Similarly, CPT1C regulates fatty acid β–oxidation in mitochondria by facilitating the transport of long-chain fatty acids into mitochondria. This process is primarily governed by the AMP–activated protein kinase (AMPK)/Acetyl–CoA Carboxylase (ACC)/CPT1C signaling pathway. Notably, a recent study demonstrated that CPT1C expression is significantly influenced by microRNA–148a–3p (miR–148a–3p) in Chinese Guizhou Congjiang Xiang pigs. Overexpression of miR–148a–3p markedly reduced the phosphorylation levels of AMPK, ACC, and CPT1C, thereby suppressing fatty acid β–oxidation and promoting lipid accumulation in preadipocytes, which in turn accelerated adipogenic differentiation. Conversely, inhibition of miR–148a–3p increased the levels of phosphorylated AMPK (p–AMPK), phosphorylated ACC (p–ACC), and CPT1C, enhancing fatty acid β–oxidation and reducing lipid deposition. Through the modulation of AMPK/ACC/CPT1C signaling axis, miR–148a–3p indirectly regulates the metabolic balance between fatty acid oxidation and lipid storage [63]. CIDEB, an ER-associated protein, also plays a pivotal role in regulating IMF accumulation in goat muscle cells by modulating lipid metabolism [56].

ER–mitochondria signaling pathways further shape IMF deposition in skeletal muscles through transcriptional regulation and stress responses. Ran et al. [64] indicated that silent mating information regulation 2 homolog 1 (SIRT1) influences IMF accumulation by interacting with three potential transcription factors, including nuclear factor erythroid 2–related factor 1, and four target genes encoded MAPK1 and hydroxyacyl–CoA dehydrogenase, which are involved in ER protein processing and the MAPK signaling pathway in yaks. Additionally, ER dysfunction—often characterized by the induction of the unfolded protein response (UPR)—can exacerbate mitochondrial dysfunction [65], contributing to metabolic imbalance that promotes IMF deposition [66]. Studies in yak and swine suggest that the ER–mitochondrial system regulates IMF deposition through the AMPK/SIRT1 signaling axis, highlighting its potential as a therapeutic target to improve muscle quality and alleviate lipid metabolic disorders in the muscle [64,67].

Insulin sensitivity also determines IMF deposition via ER–mitochondrial communication. Under insulin-resistant (IR) conditions, adipose tissue becomes less responsive to the antilipolytic effects of insulin, resulting in sustained elevation of plasma free fatty acid (FFA) levels. Skeletal muscle cells take up FFAs by fatty–acid transporters such as CD36, and subsequently re–esterify them into triglycerides, leading to the expansion of lipid droplets. This process has been observed in miniature pigs fed a high–fat, high–fructose diet. Notably, despite preserved hepatic insulin sensitivity, these miniature pigs exhibited skeletal muscle IR accompanied by substantial IMF accumulation [68]. Moreover, IR alters the expression profiles of long noncoding RNAs (lncRNAs) and microRNAs (miRNAs) involved in adipogenic gene networks. In Laiwu pigs, the induction of IR disrupted specific lncRNA–miRNA–mRNA interactions that target PPARγ and C/EBPα, thereby lifting post-transcriptional repression of adipogenic effectors and promoting ectopic lipid storage within muscle fibers [69]. Although research on how the ER–mitochondrial system affects IMF content via insulin sensitivity in livestock remains limited, relevant medical studies offer valuable insights for future research in domestic animals. For instance, under IR conditions, disrupted ER-mitochondria and mitochondria–lipid droplet contacts impair the direct channeling of fatty acids into mitochondria, leading to ectopic triacylglycerol accumulation [70]. In mice muscle fibers, Thoudam et al. [71] reported that obesity-induced upregulation of pyruvate dehydrogenase kinase 4 (PDK4) stabilizes the IP_3_R1–GRP75–VDAC1 complex at MAMs, thereby increasing the calcium flux into mitochondria. The resulting calcium overload impairs oxidative phosphorylation, elevates ROS production, activates c–Jun N–terminal kinase (JNK) signaling, and attenuates insulin-stimulated AKT signaling—ultimately reducing fatty acid oxidation and promoting lipid accumulation.

The structural integrity of ER–mitochondrial contacts is also a critical determinant of mitochondrial function and intramuscular lipid deposition. In pigs, PINK1 deficiency disrupts ER–mitochondrial tethering, impairs mitophagy and oxidative metabolism, and markedly enhances IMF accumulation. Similarly, ACE2–null mice exhibit pronounced ER stress, mitochondrial dysfunction, and elevated muscle triglycerides, whereas ACE2 activation partially alleviates ER–mitochondrial stress and reduces lipid deposition [72]. These findings collectively suggest that compromised ER–mitochondrial communication leads to a metabolic milieu favoring lipid accretion, while restoring this interface exerts a protective effect. However, findings from in vitro systems can be divergent, adding complexity to our understanding of ER–mitochondrial interactions. For example, GRP75 knockdown in 3T3–L1 adipocytes suppresses mitochondrial function and adipogenic differentiation, implying that intact MAM architecture is essential for adipocyte maturation. This outcome contrasts with observations in porcine intramuscular adipogenic progenitors, where reduced ER–mitochondrial coupling is associated with increased lipid deposition [73]. These discrepancies may arise from species–specific metabolic adaptations, distinct adipocyte lineages, or differences in molecular pathways involved (e.g., mitophagy regulation versus Ca^2+^ signaling). Despite on–going progress, the precise mechanisms linking altered ER–mitochondrial coupling to lipid accumulation—whether through dysregulated Ca^2+^ flux, impaired lipid trafficking, defective mitophagy, or redox imbalance—remain poorly defined. Furthermore, the functions of many other MAM-associated proteins in intramuscular adipogenesis are still largely unexplored. A systematic, comparative framework encompassing species, cell types, and perturbation models is therefore essential to elucidate how ER–mitochondrial communication shapes the transcriptional and metabolic programs underlying IMF deposition.

### 2.2. Muscle Fiber Type

Muscle fiber composition is an important trait of meat quality [74]. A higher proportion of oxidative fibers (Type I/IIa) is associated with redder color, better water-holding capacity, greater tenderness, and improved flavor, primarily due to increased IMF content [75]. In contrast, meat with a high proportion of glycolytic fibers (Type IIx/IIb) tends to be paler, tougher and drier [76,77,78]. Thus, optimizing the composition of muscle fiber is a crucial strategy for improving meat quality.

The intricate ER–mitochondrial crosstalk enables muscle fiber type transformation through the metabolic and calcium-dependent signaling network. In weaned pigs, cold exposure enhances mitochondrial biogenesis and respiration, promoting the transition from fast glycolytic to slow oxidative fibers [79]. In particular, overexpression of PPARγ coactivator 1–alpha (PGC–1α), a regulator of mitochondrial biogenesis, shifts muscle metabolism toward enhanced fatty acid oxidation and a greater proportion of red, oxidative fibers in both mice and pigs. Elevated PGC–1α levels directly correlate with the upregulation of oxidative fiber markers such as myosin heavy chain type I (MyHC I), and enhanced activity of the TCA cycle and electron transport chain enzymes [80]. Meanwhile, the ER serves as the primary calcium reservoir, with calcium acting as a second messenger in various signaling pathways. In muscle cells, calcium released via IP_3_R and ryanodine receptors (RyRs) from the ER ensures its precise delivery for excitation–contraction coupling. Elevated cytoplasmic calcium activates calcium/calmodulin-dependent protein kinase kinase 2 (CaMKK2), which in turn stimulates AMPK, promoting mitochondrial biogenesis and a shift toward oxidative muscle fiber types [81]. This calcium-dependent signaling cascade drives metabolic reprogramming of muscle fibers. For instance, overexpression of MyoD family inhibitor (MDFI) in C2C12 myoblasts increases intracellular calcium, enhances CaMKK2 and AMPK phosphorylation, and promotes a transition from fast to slow fiber types [81].

Substantial evidence indicates that ER–mitochondrial communication promotes oxidative fiber formation by coordinating calcium signaling, activating the CaMKK2–AMPK pathway, and enhancing PGC–1α–dependent mitochondrial biogenesis. However, the link between MAM dynamics and fiber-type regulation is more complicated than previously understood. Some studies reported reduced ER–mitochondrial contacts after endurance exercise [82], while others observed an increased contact density in glycolytic fiber–enriched muscle [83]. These discrepancies may reflect species–specific physiology, the short-term effect of metabolic stimuli, or differences between acute and chronic adaptive responses. A key unresolved question is whether MAM remodeling primarily drives fiber–type transitions or occurs in response to altered energy demand. Moreover, many MAM–resident proteins involved in fiber–type specification remain largely unidentified. Addressing these questions, particularly in livestock species, will be essential to assess whether targeting ER–mitochondrial interactions represent a viable strategy to enhance oxidative fiber composition and improve meat quality.

### 2.3. Tenderness

Postmortem muscle tenderness is a key factor in meat quality and strongly influences consumer preferences. It can be regulated by a series of biochemical processes, including muscle fiber structure, protein proteolysis, and apoptosis [84]. Therefore, optimizing postmortem muscle tenderness is vital for improving sensory attributes and marketability. Interestingly, ER–mitochondrial interactions also regulate apoptosis, protein proteolysis, and other related processes involved in postmortem muscle tenderization [85].

#### 2.3.1. The Effects and Mechanisms of the ER–Mitochondrial System on Apoptosis

The ER–mitochondrial system contributes to postmortem muscle tenderness primarily by activating apoptotic and proteolytic pathways following slaughter–induced ischemia. Mitochondria are important in regulating cellular progress linked to tenderization. Under slaughter–induced stress conditions including energy deficit and oxidative stress, mitochondrial permeability transition pores (MPTPs) open, leading to membrane potential collapse and the release of apoptogenic factors like cytochrome c into the sarcoplasm [86].

The ER–mitochondrial system also promotes postmortem muscle tenderness via caspase-dependent apoptotic pathways, as demonstrated in livestock studies. In chicken muscles, ERS caused by protein misfolding or calcium imbalance induced UPR via three key sensors: protein kinase R-like ER kinase (PERK), inositol–requiring enzyme 1, and activating transcription factor 6 (ATF6). PERK phosphorylates eIF2α, enabling the selective translation of ATF4, which activates the expression of C/EBP homologous protein (CHOP). CHOP is a transcription factor that downregulates Bcl–2 and sensitizes cells to death signals. Meanwhile, prolonged IRE1α activation recruits TRAF2 and ASK1, activates JNK, and promotes OMM permeabilization [87]. In pheochromocytoma cells, calcium released by the ER activates ER–specific caspase–12, which cleaves caspase–7 and amplifies the apoptotic signal [88,89]. Inhibition of the PERK or JNK pathways reduces cytochrome c release and downstream caspase activation, emphasizing the role of UPR-mediated signals in apoptosis [86,87,90]. In addition, mitochondria take up ER-released calcium through the calcium uniporter, leading to mitochondrial matrix calcium overload, loss of membrane potential, and opening of MPTPs. This cascade triggers cytochrome c release into the cytosol, where it forms the apoptosome after binding to apoptosis-protease activating factor 1 and activates caspase–9 [86]. Caspase–9 then cleaves and activates its effector caspase–3 and mediates cellular demolition. In postmortem yak muscle, caspase–3 cooperates with calcium–dependent calpains to degrade myofibrillar proteins and thus affects postmortem muscle tenderness [85,86,91]. Similar ER–mitochondrial interactions are also observed in broiler, where increased like GRP kinase 78 and CHOP, along with a higher pro–apoptotic effector molecules (Bax)/anti–apoptotic effector molecules (Bcl–2) ratio, activates caspase-9 and caspase–3 [90]. Additionally, the ERS–induced activation of proteolytic systems such as calpain also contributes to postmortem myolysis [92].

#### 2.3.2. The Effects and Mechanisms of the ER-Mitochondrial System on Muscle Proteolysis

Postmortem myolysis is closely associated with cysteine proteases such as calpains that are activated by rising intracellular calcium levels [93]. As central regulators to cellular calcium homeostasis, the ER and mitochondria are important for calpain activity in postmortem muscle.

The ER is the primary intracellular calcium reservoir. After cell death, SERCA inactivation causes uncontrolled calcium release into the cytoplasm [88], while postmortem hypoxia and acidosis further disrupt ER membrane integrity and ion gradients, exacerbating calcium leakage. The resulting cytoplasmic calcium surge activates μ–calpain and m–calpain, which mediate the proteolytic degradation of myofibrillar proteins [94]. Studies have shown that calpain–mediated proteolysis is significant for the degradation of key structural proteins such as desmin [95], titin, and nebulin [96], which contribute to postmortem myolysis and meat tenderization. Mitochondria also help regulate cytosolic calcium, but membrane dysfunction impairs this regulation, increasing calcium leakage in the cytosol. The opening of the MPTP induces mitochondrial swelling, membrane rupture, and the release of pro–death signals, further promoting calcium efflux and cellular disintegration [97,98]. Similar to the effect in the ER, dysfunctional mitochondria elevate cytosolic calcium levels, activate calpain, and promote proteolysis. Conversely, studies on beef and pork tissues showed reduced protein degradation when mitochondrial activities are inhibited, highlighting the crucial role of the ER–mitochondrial system in myolysis [99,100,101]. Moreover, MAMs facilitate calcium exchange between the ER and mitochondria. The disruption of these junctions after slaughter accelerates the increase in free calcium, further activating calpain and coordinating the degradation of other proteolytic systems within the muscle [88].

Emerging evidence underscores the critical role of ER–mitochondria communication in postmortem muscle tenderization, primarily through coordinating calcium flux, apoptotic signaling, and proteolytic activation. Studies in poultry, yak, and porcine muscles showed that ER stress induces cytosolic calcium overload, triggering mitochondrial dysfunction and activating μ–calpain, caspase–9, and caspase–3. These proteases accelerate myofibrillar protein degradation, thereby enhancing meat tenderization. This mechanism is further supported by quantitative phosphoproteomic analyses, which identified key mitochondrial apoptotic pathways participating in muscle tenderization [102]. Collectively, these findings suggest a synergistic model in which ER calcium release, mitochondrial permeability transition, and downstream protease activation jointly mediate postmortem myolysis. However, several inconsistencies complicate this model. In some livestock studies, suppressing mitochondrial apoptosis failed to yield corresponding changes in shear force or proteolytic activity [92]. These discrepancies may result from species-specific muscle fiber composition, differences in postmortem aging conditions, or the varying contributions of calpain–versus caspase–mediated proteolytic pathways [88]. Furthermore, the precise signaling cascade linking ER calcium efflux, MAMs remodeling, mitochondrial permeability transition, and protease activation remains poorly defined. Also, the potential crosstalk between ER–mitochondrial signaling and autophagy in postmortem proteolysis is not well understood. Addressing these gaps by comparative, species-specific studies is essential to determine whether targeting ER–mitochondrial signaling offers a reliable strategy for improving meat tenderness in commercial production.

## 3. Conclusions and Prospects

**Figure 5:**
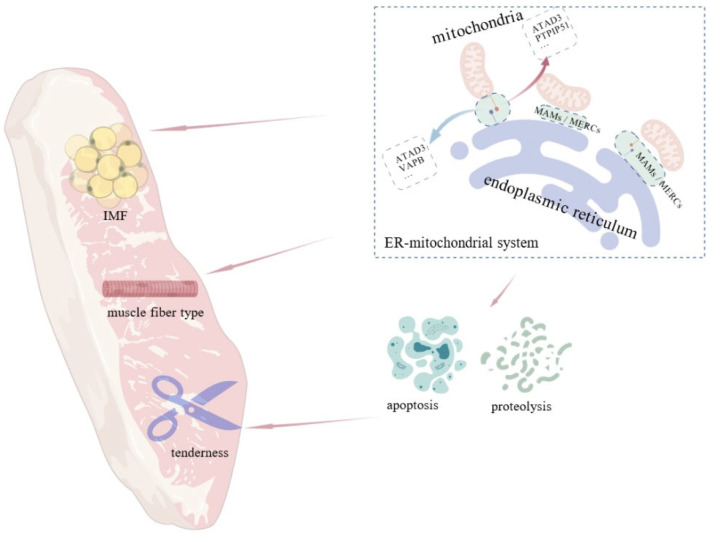
Influence of the ER–mitochondrial system on intramuscular fat, muscle fiber type, and meat tenderness. MAMs influences meat quality traits including IMF content, muscle fiber type, and tenderness by regulating crucial cellular processes like cell apoptosis and proteolysis.

Recent advances have clarified the pivotal role of ER-mitochondrial communication in regulating meat quality. Their interaction influences lipid metabolism, calcium homeostasis, and oxidative stress, thereby modulating IMF deposition, muscle fiber transformation, apoptosis, and postmortem muscle proteolysis, all of which are vital to meat quality (Figure 5). In addition, through the regulation of mitochondrial biogenesis, oxidative phosphorylation, lipid oxidation, and stress-responsive proteins, the ER–mitochondria system influences tenderness, color and water-holding capacity—all critical determinants of meat quality.

Recent studies also explored key regulators within the ER–mitochondrial system, including proteins such as PINK1, CPT1C, CIDEB, as well as non–coding RNAs like lncRNA and miRNAs, which modulate lipid synthesis and ROS production. These findings have helped identify specific signaling pathways—such as the AMPK/SIRT1 pathway—that influence metabolism and meat quality. Furthermore, nutritional interventions targeting the ER–mitochondrial axis show promise in improving meat quality.

Despite the important role of the ER–mitochondrial system in muscle, several challenges remain in harnessing its full potential to improve meat quality. First, the mechanisms by which the ER–mitochondrial system regulates metabolic pathways in muscle cells are not fully understood. In particular, the role of MAMs in calcium signaling, redox regulation, and lipid metabolism requires further clarification, including whether MAM distance correlates with metabolic progresses like lipid metabolism. Second, the complex interplay among mitochondrial dysfunction, ERS, lipid accumulation, and muscle fiber type transition warrants deeper investigation. Third, current research on species-specific differences in the ER–mitochondrial system remains limited, and it is still unclear whether these differences affect meat quality. Future studies should clarify the molecular mechanisms underlying the ER–mitochondrial regulation on meat quality, particularly the dynamic interactions with other organelles, and their contributions to cellular homeostasis. Investigating species-specific characteristics of the ER–mitochondrial system will also help optimize nutrition interventions for different livestock. Finally, as nutrition greatly influences the ER–mitochondrial function, precise nutrition approaches targeting mitochondrial biogenesis, oxidative stress reduction, and muscle fiber transition should be further explored to enhance meat quality.

In conclusion, the ER–mitochondrial system plays a pivotal role in regulating meat quality by modulating various key physiological processes. While current studies have provided valuable insights into its molecular mechanisms, future research still needs to elucidate more detailed regulatory functions of this system and its species–specific roles. Such work is essential for improving meat quality and promoting sustainable, efficient livestock production in the future.

## Data Availability

No new data were created or analyzed in this study. Data sharing is not applicable to this article.

## Supplementary Materials

The following supporting information can be downloaded at: https://www.mdpi.com/article/10.3390/ani15233465/s1, Table S1: Concise Overview of ER–Mitochondrial Signalling Pathways Relevant to Meat Quality.

## Abbreviations

The following abbreviations are used in this manuscript:

ER	endoplasmic reticulum
OMM	outer mitochondrial membrane
IMM	inner mitochondrial membrane
MM	mitochondrial matrix
IMS	IMS
IBM	inner boundary membrane
TCA	tricarboxylic acid
MAMs	mitochondria-associated membranes
MERCs	mitochondria- endoplasmic reticulum contacts
ERS	endoplasmic reticulum stress
AAA	ATPase family ATPases associated with diverse cellular activities
ATAD3	ATPase family AAA domain-containing protein 3
VAPB	vesicle-associated membrane protein B
PTPIP51	tyrosine phosphatase-interacting protein–51
SERCA	sarco/endoplasmic reticulum calcium–ATPase
VDACs	voltage–dependent anion channels
MCU	mitochondrial calcium uniporter
IP_3_R	inositol 1,4,5–trisphosphate receptors
GRP75	glucose–regulated protein 75
ROS	reactive oxygen species
TMX1	thioredoxin–related transmembrane proteins 1
Sig–1R	Sigma–1 receptor
4-HNE	4–hydroxynonenal
RIPK1	receptor–interacting protein kinase 1
RIPK3	receptor–interacting protein kinase 3
MLKL	mixed lineage kinase domain–like protein
PINK1	phosphatase and tensin homolog-induced kinase 1
CPT1C	palmitoyltransferase 1C
DFF45	DNA fragmentation factor 45
CIDEB	cell death–inducing DNA fragmentation factor 45–like effector b
AMPK	AMP–activated protein kinase
ACC	Acetyl–CoA Carboxylase
miR–148a–3p	microRNA–148a–3p
Phosphorylated Acetyl–CoA Carboxylase	p–ACC
SIRT1	silent mating information regulation 2 homolog 1
UPR	unfolded protein response
IR	insulin–resistant
FFA	free fatty acid
lncRNAs	long noncoding RNAs
miRNAs	microRNAs
PDK4	pyruvate dehydrogenase kinase 4
JNK	c–Jun N–terminal kinase
PGC–1α	PPARγ coactivator 1-alpha
MyHC I	myosin heavy chain type I
RyRs	ryanodine receptors
CaMKK2	calcium/calmodulin-dependent protein kinase kinase 2
MDFI	MyoD family inhibitor
MPTPs	mitochondrial permeability transition pores
PERK	protein kinase R–like ER kinase
ATF6	activating transcription factor 6
CHOP	C/EBP homologous protein

## References

[1] Harrington J.S., Ryter S.W., Plataki M., Price D.R., Choi A.M.K. (2023). Mitochondria in health, disease, and aging. Physiol. Rev..

[2] Teixeira P., Galland R., Chevrollier A. (2024). Super-resolution microscopies, technological breakthrough to decipher mitochondrial structure and dynamic. Semin. Cell Dev. Biol..

[3] Wikstrom M., Pecorilla C., Sharma V. (2023). The mitochondrial respiratory chain. Enzymes.

[4] Suomalainen A., Nunnari J. (2024). Mitochondria at the crossroads of health and disease. Cell.

[5] Celik C., Lee S.Y.T., Yap W.S., Thibault G. (2023). Endoplasmic reticulum stress and lipids in health and diseases. Prog. Lipid Res..

[6] Wiseman R.L., Mesgarzadeh J.S., Hendershot L.M. (2022). Reshaping endoplasmic reticulum quality control through the unfolded protein response. Mol. Cell.

[7] Coscia S.M., Thompson C.P., Tang Q., Baltrusaitis E.E., Rhodenhiser J.A., Quintero-Carmona O.A., Lakadamyali M., Holzbaur E.L.F. (2023). Myo19 tethers mitochondria to endoplasmic reticulum-associated actin to promote mitochondrial fission. J. Cell Sci..

[8] Gatti P., Schiavon C., Cicero J., Manor U., Germain M. (2025). Mitochondria- and ER-associated actin are required for mitochondrial fusion. Nat. Commun..

[9] Zhao W.B., Sheng R. (2025). The correlation between mitochondria-associated endoplasmic reticulum membranes (MAMs) and Ca^2+^ transport in the pathogenesis of diseases. Acta Pharmacol. Sin..

[10] Csordás G., Weaver D., Hajnóczky G. (2018). Endoplasmic Reticulum-Mitochondrial Contactology: Structure and Signaling Functions. Trends Cell Biol..

[11] Shirokova O.M., Pchelin P.V., Mukhina I.V. (2020). MERCs. The Novel Assistant to Neurotransmission?. Front. Neurosci..

[12] Cheng H., Gang X.K., He G.Y., Liu Y.J., Wang Y.X., Zhao X., Wang G.X. (2020). The Molecular Mechanisms Underlying Mitochondria-Associated Endoplasmic Reticulum Membrane-Induced Insulin Resistance. Front. Endocrinol..

[13] Sathyamurthy V.H., Nagarajan Y., Parvathi V.D. (2024). Mitochondria-Endoplasmic Reticulum Contact Sites (MERCS): A New Axis in Neuronal Degeneration and Regeneration. Mol. Neurobiol..

[14] Lee S., Min K.T. (2018). The Interface Between ER and Mitochondria: Molecular Compositions and Functions. Mol. Cells.

[15] Baudier J. (2018). ATAD3 proteins: Brokers of a mitochondria-endoplasmic reticulum connection in mammalian cells. Biol. Rev..

[16] Mórotz G.M., Martín-Guerrero S.M., Markovinovic A., Paillusson S., Russell M.R.G., Machado P.M.P., Fleck R.A., Noble W., Miller C.C.J. (2022). The PTPIP51 coiled-coil domain is important in VAPB binding, formation of ER-mitochondria contacts and IP3 receptor delivery of Ca2+ to mitochondria. Front. Cell Dev. Biol..

[17] Wang X.L., Wen Y.J., Dong J., Cao C.C., Yuan S.Q. (2018). Systematic In-Depth Proteomic Analysis of Mitochondria-Associated Endoplasmic Reticulum Membranes in Mouse and Human Testes. Proteomics.

[18] Vance J.E. (1990). Phospholipid synthesis in a membrane fraction associated with mitochondria. J. Biol. Chem..

[19] Rizzuto R., Pinton P., Carrington W., Fay F.S., Fogarty K.E., Lifshitz L.M., Tuft R.A., Pozzan T. (1998). Close contacts with the endoplasmic reticulum as determinants of mitochondrial Ca^2+^ responses. Science.

[20] Csordas G., Thomas A.P., Hajnoczky G. (1999). Quasi-synaptic calcium signal transmission between endoplasmic reticulum and mitochondria. EMBO J..

[21] Watanabe S., Murata Y., Oka Y., Oiwa K., Horiuchi M., Iguchi Y., Komine O., Sobue A., Katsuno M., Ogi T. (2023). Mitochondria- associated membrane collapse impairs TBK1-mediated proteostatic stress response in ALS. Proc. Natl. Acad. Sci. USA.

[22] Li Y.E., Sowers J.R., Hetz C., Ren J. (2022). Cell death regulation by MAMs: From molecular mechanisms to therapeutic implications in cardiovascular diseases. Cell Death Dis..

[23] Flis V.V., Daum G. (2013). Lipid Transport between the Endoplasmic Reticulum and Mitochondria. Cold Spring Harb. Perspect. Biol..

[24] Schwarz L., Sharma K., Dodi L.D., Rieder L.S., Fallier-Becker P., Casadei N., Fitzgerald J.C. (2022). Miro1 R272Q disrupts mitochondrial calcium handling and neurotransmitter uptake in dopaminergic neurons. Front. Mol. Neurosci..

[25] Peng J.J., Ran Y.Q., Xie H.J., Deng L., Li C.F., Ling C. (2021). Sarco/Endoplasmic Reticulum Ca^2+^-Transporting ATPase (SERCA) Modulates Autophagic, Inflammatory, and Mitochondrial Responses during Influenza A Virus Infection in Human Lung Cells. J. Virol..

[26] Zhang W., Ye F.H., Pang N., Kessi M., Xiong J., Chen S.M., Peng J., Yang L., Yin F. (2022). Restoration of Sarco/Endoplasmic Reticulum Ca^2+^-ATPase Activity Functions as a Pivotal Therapeutic Target of Anti-Glutamate-Induced Excitotoxicity to Attenuate Endoplasmic Reticulum Ca^2+^ Depletion. Front. Pharmacol..

[27] Zahedi R.P., Sickmann A., Boehm A.M., Winkler C., Zufall N., Schonfisch B., Guiard B., Pfanner N., Meisinger C. (2006). Proteomic analysis of the yeast mitochondrial outer membrane reveals accumulation of a subclass of preproteins. Mol. Biol. Cell.

[28] Varughese J.T., Buchanan S.K., Pitt A.S. (2021). The Role of Voltage-Dependent Anion Channel in Mitochondrial Dysfunction and Human Disease. Cells.

[29] Liu Y., Jin M.P., Wang Y.Y., Zhu J.J., Tan R., Zhao J., Ji X.Y., Jin C., Jia Y.F., Ren T.T. (2020). MCU-induced mitochondrial calcium uptake promotes mitochondrial biogenesis and colorectal cancer growth. Signal Transduct. Target. Ther..

[30] Peng Y.C., Zhou L., Jin Y.J., Wu D.L., Chen N., Zhang C.C., Liu H.P., Li C.L., Ning R., Yang X.C. (2025). Calcium bridges built by mitochondria-associated endoplasmic reticulum membranes: Potential targets for neural repair in neurological diseases. Neural Regen. Res..

[31] Atakpa-Adaji P., Ivanova A. (2023). IP3R at ER-Mitochondrial Contact Sites: Beyond the IP3R-GRP75-VDAC1 Ca^2+^ Funnel. Contact.

[32] Xu H., Guan N., Ren Y.L., Wei Q.J., Tao Y.H., Yang G.S., Liu X.Y., Bu D.F., Zhang Y., Zhu S.N. (2018). IP3R-Grp75-VDAC1-MCU calcium regulation axis antagonists protect podocytes from apoptosis and decrease proteinuria in an Adriamycin nephropathy rat model. BMC Nephrol..

[33] Ma X.Y., Li M.Y., Liu Y., Zhang X.F., Yang X.Y., Wang Y., Li Y.P., Wang J.Y., Liu X.H., Yan Z.Z. (2024). ARTC1-mediated VAPB ADP-ribosylation regulates calcium homeostasis. J. Mol. Cell Biol..

[34] Lennicke C., Cochemé H.M. (2021). Redox metabolism: ROS as specific molecular regulators of cell signaling and function. Mol. Cell.

[35] Sahoo B.M., Banik B.K., Borah P., Jain A. (2022). Reactive Oxygen Species (ROS): Key Components in Cancer Therapies. Anti-Cancer Agents Med. Chem..

[36] Victor P., Sarada D., Ramkumar K.M. (2021). Crosstalk between endoplasmic reticulum stress and oxidative stress: Focus on protein disulfide isomerase and endoplasmic reticulum oxidase 1. Eur. J. Pharmacol..

[37] Hao W.H., Shan W.J., Wan F., Luo J.Y., Niu Y.Y., Zhou J., Zhang Y.U., Xu N.H., Xie W.D. (2023). Canagliflozin Delays Aging of HUVECs Induced by Palmitic Acid via the ROS/p38/JNK Pathway. Antioxidants.

[38] Guo X., Zhang B.R., Chen Y.T., Jia Z., Yuan X.Y., Zhang L., Liu J., Liu Y.A. (2025). Multifunctional mesoporous nanoselenium delivery of metformin breaks the vicious cycle of neuroinflammation and ROS, promotes microglia regulation and alleviates Alzheimer’s disease. Colloids Surf. B Biointerfaces.

[39] Matsuo Y. (2022). Introducing Thioredoxin-Related Transmembrane Proteins: Emerging Roles of Human TMX and Clinical Implications. Antioxid. Redox Signal..

[40] Guo Z.Y., Yu X.T., Zhao S., Zhong X., Huang D., Feng R.Y., Li P., Fang Z.Y., Hu Y.Q., Zhang Z.T. (2023). SIRT6 deficiency in endothelial cells exacerbates oxidative stress by enhancing HIF1α accumulation and H3K9 acetylation at the Ero1α promoter. Clin. Transl. Med..

[41] Lebiedzinska-Arciszewska M., Pakula B., Bonora M., Missiroli S., Potes Y., Jakubek-Olszewska P., Simoes I.C.M., Pinton P., Wieckowski M.R. (2024). Distribution of the p66Shc Adaptor Protein Among Mitochondrial and Mitochondria-Associated Membranes Fractions in Normal and Oxidative Stress Conditions. Int. J. Mol. Sci..

[42] Lebiedzinska-Arciszewska M., Oparka M., Vega-Naredo I., Karkucinska-Wieckowska A., Pinton P., Duszynski J., Wieckowski M.R. (2015). The interplay between p66Shc, reactive oxygen species and cancer cell metabolism. Eur. J. Clin. Investig..

[43] Li Z.X., Ran Q., Qu C., Hu S., Cui S.Y., Zhou Y., Shen B., Yang B. (2025). Sigma-1 receptor activation attenuates DOX-induced cardiotoxicity by alleviating endoplasmic reticulum stress and mitochondrial calcium overload via PERK and IP3R-VDAC1-MCU signaling pathways. Biol. Direct.

[44] Wang B.Q., Wang Y., Zhang J., Hu C., Jiang J., Li Y.M., Peng Z.Y. (2023). ROS-induced lipid peroxidation modulates cell death outcome: Mechanisms behind apoptosis, autophagy, and ferroptosis. Arch. Toxicol..

[45] Li F., Guan Z., Gao Y.Y., Bai Y., Zhan X.Y., Ji X.Y., Xu J., Zhou H.M., Rao Z.Q. (2024). ER stress promotes mitochondrial calcium overload and activates the ROS/NLRP3 axis to mediate fatty liver ischemic injury. Hepatol. Commun..

[46] Montesinos J., Area-Gomez E. (2021). Lipidome changes due to accumulation of cholesterol via APP-C99 alters neuronal permeability. Alzheimer’s Dement. J. Alzheimer’s Assoc..

[47] Yeo A.J., Chong K.L., Gatei M., Zou D.X., Stewart R., Withey S., Wolvetang E., Parton R.G., Brown A.D., Kastan M.B. (2021). Impaired endoplasmic reticulum-mitochondrial signaling in ataxia-telangiectasia. iScience.

[48] Mustafa M., Ahmad R., Tantry I.Q., Ahmad W., Siddiqui S., Alam M., Abbas K., Hassan M.I., Habib S., Islam S. (2024). Apoptosis: A Comprehensive Overview of Signaling Pathways, Morphological Changes, and Physiological Significance and Therapeutic Implications. Cells.

[49] Liu S.Z., Yao S.J., Yang H., Liu S.J., Wang Y.J. (2023). Autophagy: Regulator of cell death. Cell Death Dis..

[50] D’Arcy M.S. (2019). Cell death: A review of the major forms of apoptosis, necrosis and autophagy. Cell Biol. Int..

[51] Newton K., Strasser A., Kayagaki N., Dixit V.M. (2024). Cell death. Cell.

[52] Lawen A. (2003). Apoptosis-an introduction. Bioessays.

[53] Klionsky D.J. (2005). Autophagy. Curr. Biol..

[54] Li D., Meng L., Xu T., Su Y., Liu X., Zhang Z., Wang X. (2017). RIPK1-RIPK3-MLKL-dependent necrosis promotes the aging of mouse male reproductive system. Elife.

[55] Man S.M., Karki R., Kanneganti T.D. (2017). Molecular mechanisms and functions of pyroptosis, inflammatory caspases and inflammasomes in infectious diseases. Immunol. Rev..

[56] Huang Z., Li Q., Yang C., Zhang C., Huang L., Lin Y., Wang Y., Xiang H., Zhu J. (2025). CIDEB promotes lipid deposition in goat intramuscular adipocytes. Anim. Biosci..

[57] Zou B., Wang H., Duan M., Sun Y., Liu Y., Li X., Dai R. (2024). Identifying the Potential Apoptotic Metabolites in Postmortem Beef Muscle by Targeted Metabolomics. J. Agric. Food Chem..

[58] Zeng F., Cao J., Li W., Zhou Y., Yuan X. (2024). FNIP1: A key regulator of mitochondrial function. Biomed. Pharmacother. Biomed. Pharmacother..

[59] Cao J.X., Li N.N., Huang R.L., Jia F.J., He Z.Y., Han W.L., Liu W.Z., Li S.Q., Wang W.Y., Ren W.Y. (2025). PINK1 link mitochondria-ER contacts controls deposition of intramuscular fat in pigs. Biochem. Biophys. Res. Commun..

[60] Chen X., Wang Q., Li S., Li X.J., Yang W. (2022). Mitochondrial-Dependent and Independent Functions of PINK1. Front Cell Dev Biol.

[61] Gautier C.A., Kitada T., Shen J. (2008). Loss of PINK1 causes mitochondrial functional defects and increased sensitivity to oxidative stress. Proc. Natl. Acad. Sci. USA.

[62] Chen D.W., Ran D., Wang C., Liu Y.Y., Ma Y.G., Song R.L., Gao Y.S., Liu Z.P. (2021). Role of mitochondrial dysfunction and PINK1/Parkin-mediated mitophagy in Cd-induced hepatic lipid accumulation in chicken embryos. Life Sci..

[63] Tan L., Chen Z., Ruan Y., Xu H. (2022). Differential regulatory roles of microRNAs during intramuscular adipogenesis in Chinese Guizhou Congjiang Xiang pigs. Epigenetics.

[64] Ran H., He Q., Han Y., Wang J., Wang H., Yue B., Zhang M., Chai Z., Cai X., Zhong J. (2023). Functional study and epigenetic targets analyses of SIRT1 in intramuscular preadipocytes via ChIP-seq and mRNA-seq. Epigenetics.

[65] Deng P., Haynes C.M. (2017). Mitochondrial dysfunction in cancer: Potential roles of ATF5 and the mitochondrial UPR. Semin. Cancer Biol..

[66] Tian Q., Lee P.R., Yang Q., Moore A.Z., Landman B.A., Resnick S.M., Ferrucci L. (2024). The mediation roles of intermuscular fat and inflammation in muscle mitochondrial associations with cognition and mobility. J. Cachexia Sarcopenia Muscle.

[67] Guo Z., Chen X., Huang Z., Chen D., Li M., Yu B., He J., Luo Y., Yan H., Zheng P. (2022). Dihydromyricetin improves meat quality and promotes skeletal muscle fiber type transformations via AMPK signaling in growing-finishing pigs. Food Funct..

[68] Hsu M.C., Wang M.E., Jiang Y.F., Liu H.C., Chen Y.C., Chiu C.H. (2017). Long-term feeding of high-fat plus high-fructose diet induces isolated impaired glucose tolerance and skeletal muscle insulin resistance in miniature pigs. Diabetol. Metab. Syndr..

[69] Feng H., Liu T., Yousuf S., Zhang X., Huang W., Li A., Xie L., Miao X. (2022). Identification and analysis of lncRNA, miRNA and mRNA related to subcutaneous and intramuscular fat in Laiwu pigs. Front. Endocrinol..

[70] Keenan S.N., Watt M.J., Montgomery M.K. (2020). Inter-organelle Communication in the Pathogenesis of Mitochondrial Dysfunction and Insulin Resistance. Curr. Diabetes Rep..

[71] Thoudam T., Ha C.M., Leem J., Chanda D., Park J.S., Kim H.J., Jeon J.H., Choi Y.K., Liangpunsakul S., Huh Y.H. (2019). PDK4 Augments ER-Mitochondria Contact to Dampen Skeletal Muscle Insulin Signaling During Obesity. Diabetes.

[72] Cao X., Lu X.-M., Tuo X., Liu J.-Y., Zhang Y.-C., Song L.-N., Cheng Z.-Q., Yang J.-K., Xin Z. (2019). Angiotensin-converting enzyme 2 regulates endoplasmic reticulum stress and mitochondrial function to preserve skeletal muscle lipid metabolism. Lipids Health Dis..

[73] Wang C.-H., Wang C.-H., Hung P.-J., Wei Y.-H. (2022). Disruption of mitochondria-associated ER membranes impairs insulin sensitivity and thermogenic function of adipocytes. Front. Cell Dev. Biol..

[74] Zhou X., Liu Y., Zhang L., Kong X., Li F. (2021). Serine-to-glycine ratios in low-protein diets regulate intramuscular fat by affecting lipid metabolism and myofiber type transition in the skeletal muscle of growing-finishing pigs. Anim. Nutr..

[75] He Y., Hu H., Liang X., Liang J., Li F., Zhou X. (2025). Gut microbes-muscle axis in muscle function and meat quality. Sci. China Life Sci..

[76] Park J., Song S., Cheng H., Im C., Jung E.Y., Moon S.S., Choi J., Hur S.J., Joo S.T., Kim G.D. (2022). Comparison of Meat Quality and Muscle Fiber Characteristics between Porcine Skeletal Muscles with Different Architectures. Food Sci. Anim. Resour..

[77] Weng K., Huo W., Li Y., Zhang Y., Zhang Y., Chen G., Xu Q. (2022). Fiber characteristics and meat quality of different muscular tissues from slow- and fast-growing broilers. Poult. Sci..

[78] Mo M., Zhang Z., Wang X., Shen W., Zhang L., Lin S. (2023). Molecular mechanisms underlying the impact of muscle fiber types on meat quality in livestock and poultry. Front. Vet. Sci..

[79] Yu J., Chen S., Zeng Z., Xing S., Chen D., Yu B., He J., Huang Z., Luo Y., Zheng P. (2021). Effects of Cold Exposure on Performance and Skeletal Muscle Fiber in Weaned Piglets. Animals.

[80] Zhang L., Zhou Y., Wu W., Hou L., Chen H., Zuo B., Xiong Y., Yang J. (2017). Skeletal Muscle-Specific Overexpression of PGC-1alpha Induces Fiber-Type Conversion through Enhanced Mitochondrial Respiration and Fatty Acid Oxidation in Mice and Pigs. Int. J. Biol. Sci..

[81] Lu T., Zhu Y., Guo J., Mo Z., Zhou Q., Hu C.Y., Wang C. (2023). MDFI regulates fast-to-slow muscle fiber type transformation via the calcium signaling pathway. Biochem. Biophys. Res. Commun..

[82] Merle A., Jollet M., Britto F.A., Goustard B., Bendridi N., Rieusset J., Ollendorff V., Favier F.B. (2019). Endurance exercise decreases protein synthesis and ER-mitochondria contacts in mouse skeletal muscle. J. Appl. Physiol..

[83] Nieblas B., Pérez-Treviño P., García N. (2022). Role of mitochondria-associated endoplasmic reticulum membranes in insulin sensitivity, energy metabolism, and contraction of skeletal muscle. Front. Mol. Biosci..

[84] Maltin C., Balcerzak D., Tilley R., Delday M. (2003). Determinants of meat quality: Tenderness. Proc. Nutr. Soc..

[85] Zhao Y., Xiang C., Roy B.C., Bruce H.L., Blecker C., Zhang Y., Liu C., Zhang D., Chen L., Huang C. (2025). Apoptosis and its role in postmortem meat tenderness: A comprehensive review. Meat Sci..

[86] Wang L., Ma G., Zhang Y., Shi X., Han L., Yu Q., Zhao S., Ma J. (2018). Effect of mitochondrial cytochrome c release and its redox state on the mitochondrial-dependent apoptotic cascade reaction and tenderization of yak meat during postmortem aging. Food Res. Int..

[87] Xiang X., Yan N., Wang Y., Jia M., Feng X., Chen L. (2024). Mitochondrial and endoplasmic reticulum stress pathways regulate tenderness of post-mortem chicken muscle. Food Biosci..

[88] Huang F., Ding Z., Chen J., Guo B., Wang L., Liu C., Liu C., Zhang C. (2025). Contribution of mitochondria to postmortem muscle tenderization: A review. Crit. Rev. Food Sci. Nutr..

[89] Martinez J.A., Zhang Z., Svetlov S.I., Hayes R.L., Wang K.K., Larner S.F. (2010). Calpain and caspase processing of caspase-12 contribute to the ER stress-induced cell death pathway in differentiated PC12 cells. Apoptosis.

[90] Chai Y., Chen L., Xiang S., Wu L., Liu X., Luo J., Feng X. (2024). Endoplasmic reticulum stress improved chicken tenderness, promoted apoptosis and autophagy during postmortem ageing. Food Sci. Hum. Wellness.

[91] Li X., Hu L., Zhu X., Guo X., Deng X., Zhang J. (2022). The effect of caspase-3 in mitochondrial apoptosis activation on degradation of structure proteins of Esox lucius during postmortem storage. Food Chem..

[92] Ma Y., Wang Y., Wang Z., Xie Y., Tang C., Li C., Xu F., Zhou H., Xu B. (2024). New perspective for Calpain-Mediated regulation of meat Quality: Unveiling the impact on mitochondrial pathway apoptosis in post-mortem. Food Chem..

[93] Bhat Z.F., Morton J.D., Mason S.L., Bekhit A.E.-D.A. (2018). Role of calpain system in meat tenderness: A review. Food Sci. Hum. Wellness.

[94] Huang M., Huang F., Ma H., Xu X., Zhou G. (2012). Preliminary study on the effect of caspase-6 and calpain inhibitors on postmortem proteolysis of myofibrillar proteins in chicken breast muscle. Meat Sci..

[95] Chen F., Chang R., Trivedi M., Capetanaki Y., Cryns V.L. (2003). Caspase proteolysis of desmin produces a dominant-negative inhibitor of intermediate filaments and promotes apoptosis. J. Biol. Chem..

[96] Koohmaraie M. (1996). Biochemical factors regulating the toughening and tenderization processes of meat. Meat Sci..

[97] Sierra V., Olivan M. (2013). Role of mitochondria on muscle cell death and meat tenderization. Recent Pat. Endocr. Metab. Immune Drug Discov..

[98] Zhang J., Ma G., Guo Z., Yu Q., Han L., Han M., Zhu Y. (2018). Study on the apoptosis mediated by apoptosis-inducing-factor and influencing factors of bovine muscle during postmortem aging. Food Chem..

[99] Bu X., Wang H., Wang Y., Ojangba T., Nan H., Zhang L., Yu Q. (2023). Effects of iron-catalyzed oxidation and methemoglobin oxidation systems on endogenous enzyme activity and myofibrillar protein degradation in yak meat. Food Chem..

[100] Chen L., Feng X.C., Lu F., Xu X.L., Zhou G.H., Li Q.Y., Guo X.Y. (2011). Effects of camptothecin, etoposide and Ca^2+^ on caspase-3 activity and myofibrillar disruption of chicken during postmortem ageing. Meat Sci..

[101] Dang D.S., Buhler J.F., Davis H.T., Thornton K.J., Scheffler T.L., Matarneh S.K. (2020). Inhibition of mitochondrial calcium uniporter enhances postmortem proteolysis and tenderness in beef cattle. Meat Sci..

[102] Zhang J., Wang S., Ge W. (2022). Mechanisms of Mitochondrial Apoptosis-Mediated Meat Tenderization Based on Quantitative Phosphoproteomic Analysis. Foods.

