# Supplementary material for: Crosstalk Between the Endoplasmic Reticulum and Mitochondria in Skeletal Muscle: Implications for Meat Quality
Source: Animals (Basel). 2025 Dec 1;15(23):3465. doi: 10.3390/ani15233465 (PMC12691412; doi:10.3390/ani15233465)
Supplement: Supplementary file 1 [file animals-15-03465-s001.zip › animals-3994042-supplementary.pdf]

Table S1. Concise Overview of ER–Mitochondrial Signalling Pathways Relevant to Meat Quality.

Signalling Pathway	Demonstrated Roles (Evidence-Based)	Hypothesised Roles	Related Meat Traits	Species with Supporting Data
AMPK Pathway	Regulates CPT1C phosphorylation promoting β-oxidation[1]<br>Cold exposure and nutritional interventions activate AMPK to increase oxidative fibers[2]	AMPK–MAM coupling may stabilize ER–mitochondria tethering<br>May coordinate lipid oxidation and reduced ER stress	IMF, Muscle fiber type (↑ oxidative), Tenderness (via energy metabolism)	Pig[1], Yak[3], Broiler[4]
SIRT1 Pathway	Modulates IMF through MAPK1 and lipid metabolism genes[3]<br>Regulates ER protein processing and mitochondrial function	May mediate ER–mitochondria communication in response to nutritional signals<br>Potential regulator of oxidative fiber transformation	IMF, Muscle fiber type	Yak[3], Pig
PPARγ Pathway	Key regulator of adipogenic gene expression in muscle<br>Influenced by lncRNA–miRNA networks (e.g., miR-148a-3p–CPT1C axis)[5]	ER–mitochondria dysfunction may shift balance toward PPARγ-driven adipogenesis	IMF deposition	Pig, Goat[6]
Ca^{2+}–IP_3R/VDAC1 /MCU Axis	Mediates ER→mitochondria Ca^{2+} transfer[7]<br>Excess Ca^{2+} triggers mitochondrial apoptosis (cytochrome c release)[5]	MAM distance may determine Ca^{2+} microdomain strength affecting metabolism and apoptosis	Tenderness (postmortem apoptosis), IMF regulation	Pig[8], Chicken[9], Yak[10]
CaMKK2–AMPK Signalling	Ca^{2+}-dependent activation promotes oxidative fiber formation<br>MDFI overexpression → Ca^{2+}↑ → CaMKK2–AMPK↑ → slow fiber transition[11]	May coordinate ER Ca^{2+} release with mitochondrial remodeling	Muscle fiber type (↑ oxidative fibers)	Mouse, Pig[12]
UPR (PERK–ATF4–CHOP, IRE1–JNK)	ER stress triggers CHOP ↑, Bcl-2↓, caspase activation → apoptosis[9]	Chronic mild ER stress may modulate IMF via altered Ca^{2+} flux and ROS	Tenderness (postmortem proteolysis), IMF	Chicken, Yak[10]
ROS / p66Shc Signalling	ROS generated at MAMs influences Ca^{2+} channels and mitochondrial dysfunction[10]<br>p66Shc amplifies H_2O_2 → enhances apoptosis[13]	ROS-mediated MAM remodeling may alter fiber type and lipid metabolism	Tenderness, Muscle fiber type	Yak, Chicken[9]
PDK4–JNK–Akt Axis	Obesity-induced PDK4 stabilizes IP_3R–GRP75–VDAC1 complex → mitochondrial Ca^{2+} overload → insulin resistance[14]	ER–mitochondria Ca^{2+} transport may determine lipid partitioning in muscle	IMF deposition (via insulin sensitivity)	Mouse; livestock[8,15]

References

1. Tan, L.; Chen, Z.; Ruan, Y.; Xu, H. Differential regulatory roles of microRNAs during intramuscular adipogenesis in Chinese Guizhou Congjiang Xiang pigs. *Epigenetics* **2022**, *17*, 1800-1819, doi:10.1080/15592294.2022.2086675.
2. Yu, J.; Chen, S.; Zeng, Z.; Xing, S.; Chen, D.; Yu, B.; He, J.; Huang, Z.; Luo, Y.; Zheng, P.; et al. Effects of Cold Exposure on Performance and Skeletal Muscle Fiber in Weaned Piglets. *Animals (Basel)* **2021**, *11*, doi:10.3390/ani11072148.
3. Ran, H.; He, Q.; Han, Y.; Wang, J.; Wang, H.; Yue, B.; Zhang, M.; Chai, Z.; Cai, X.; Zhong, J.; et al. Functional study and epigenetic targets analyses of SIRT1 in intramuscular preadipocytes via ChIP-seq and mRNA-seq. *Epigenetics* **2023**, *18*, 2135194, doi:10.1080/15592294.2022.2135194.
4. Weng, K.; Huo, W.; Li, Y.; Zhang, Y.; Zhang, Y.; Chen, G.; Xu, Q. Fiber characteristics and meat quality of different muscular tissues from slow- and fast-growing broilers. *Poultry science* **2022**, *101*, 101537, doi:10.1016/j.psj.2021.101537.
5. Feng, H.; Liu, T.; Yousuf, S.; Zhang, X.; Huang, W.; Li, A.; Xie, L.; Miao, X. Identification and analysis of lncRNA, miRNA and mRNA related to subcutaneous and intramuscular fat in Laiwu pigs. *Frontiers in endocrinology* **2022**, *13*, 1081460, doi:10.3389/fendo.2022.1081460.
6. Huang, Z.; Li, Q.; Yang, C.; Zhang, C.; Huang, L.; Lin, Y.; Wang, Y.; Xiang, H.; Zhu, J. CIDEB promotes lipid deposition in goat intramuscular adipocytes. *Anim Biosci* **2025**, doi:10.5713/ab.24.0584.
7. Atakpa-Adaji, P.; Ivanova, A. IP3R at ER-Mitochondrial Contact Sites: Beyond the IP3R-GRP75-VDAC1 Ca2+ Funnel. *Contact (Thousand Oaks (Ventura County, Calif.))* **2023**, *6*, 25152564231181020, doi:10.1177/25152564231181020.
8. Cao, J.X.; Li, N.N.; Huang, R.L.; Jia, F.J.; He, Z.Y.; Han, W.L.; Liu, W.Z.; Li, S.Q.; Wang, W.Y.; Ren, W.Y.; et al. PINK1 link mitochondria-ER contacts controls deposition of intramuscular fat in pigs. *Biochemical and Biophysical Research Communications* **2025**, *759*, doi:10.1016/j.bbrc.2025.151672.
9. Xiang, X.; Yan, N.; Wang, Y.; Jia, M.; Feng, X.; Chen, L. Mitochondrial and endoplasmic reticulum stress pathways regulate tenderness of post-mortem chicken muscle. *Food Bioscience* **2024**, *60*, doi:10.1016/j.fbio.2024.104321.
10. Wang, L.; Ma, G.; Zhang, Y.; Shi, X.; Han, L.; Yu, Q.; Zhao, S.; Ma, J. Effect of mitochondrial cytochrome c release and its redox state on the mitochondrial-dependent apoptotic cascade reaction and tenderization of yak meat during postmortem aging. *Food Res Int* **2018**, *111*, 488-497, doi:10.1016/j.foodres.2018.05.049.
11. Lu, T.; Zhu, Y.; Guo, J.; Mo, Z.; Zhou, Q.; Hu, C.Y.; Wang, C. MDFI regulates fast-to-slow muscle fiber type transformation via the calcium signaling pathway. *Biochem Biophys Res Commun* **2023**, *671*, 215-224, doi:10.1016/j.bbrc.2023.05.053.
12. Zhang, L.; Zhou, Y.; Wu, W.; Hou, L.; Chen, H.; Zuo, B.; Xiong, Y.; Yang, J. Skeletal Muscle-Specific Overexpression of PGC-1alpha Induces Fiber-Type Conversion through Enhanced Mitochondrial Respiration and Fatty Acid Oxidation in Mice and Pigs. *Int J Biol Sci* **2017**, *13*, 1152-1162, doi:10.7150/ijbs.20132.
13. Lebiedzinska-Arciszewska, M.; Pakula, B.; Bonora, M.; Missiroli, S.; Potes, Y.; Jakubek-Olszewska, P.; Simoes, I.C.M.; Pinton, P.; Wieckowski, M.R. Distribution of the p66Shc Adaptor Protein Among Mitochondrial and Mitochondria-Associated Membranes Fractions in Normal and Oxidative Stress Conditions. *International journal of molecular sciences* **2024**, *25*, doi:10.3390/ijms252312835.

14. Thoudam, T.; Ha, C.M.; Leem, J.; Chanda, D.; Park, J.S.; Kim, H.J.; Jeon, J.H.; Choi, Y.K.; Liangpunsakul, S.; Huh, Y.H.; et al. PDK4 Augments ER-Mitochondria Contact to Dampen Skeletal Muscle Insulin Signaling During Obesity. *Diabetes* **2019**, *68*, 571-586, doi:10.2337/db18-0363.
15. Cao, X.; Lu, X.-M.; Tuo, X.; Liu, J.-Y.; Zhang, Y.-C.; Song, L.-N.; Cheng, Z.-Q.; Yang, J.-K.; Xin, Z. Angiotensin-converting enzyme 2 regulates endoplasmic reticulum stress and mitochondrial function to preserve skeletal muscle lipid metabolism. *Lipids in Health and Disease* **2019**, *18*, doi:10.1186/s12944-019-1145-x.
